# No Effects of Omega-3 Supplementation on Kynurenine Pathway, Inflammation, Depressive Symptoms, and Stress Response in Males: A Placebo-Controlled Trial

**DOI:** 10.3390/nu16213744

**Published:** 2024-10-31

**Authors:** Monika Bidzan-Wiącek, Maja Tomczyk, Magdalena Błażek, Adriana Mika, Jędrzej Antosiewicz

**Affiliations:** 1 Department of Bioenergetics and Physiology of Exercise, Medical University of Gdansk, 80-210 Gdansk, Poland; jedrzej.antosiewicz@gumed.edu.pl; 2 Department of Biochemistry, University of Physical Education and Sport, 80-336 Gdansk, Poland; maja.tomczyk@awf.gda.pl; 3 Department of Quality of Life Research, Medical University of Gdansk, 80-210 Gdansk, Poland; magdalena.blazek@gumed.edu.pl; 4 Department of Environmental Analytics, Faculty of Chemistry, University of Gdansk, 80-308 Gdansk, Poland; adriana.mika@ug.edu.pl; 5 Department of Pharmaceutical Biochemistry, Medical University of Gdansk, 80-211 Gdansk, Poland

**Keywords:** kynurenine pathway, omega-3 supplementation, *n*-3 polyunsaturated fatty acid, depressive symptoms, mood, stress induction

## Abstract

**Background:** Increased inflammation and heightened physiological stress reactivity have been associated with pathophysiology of depressive symptoms. The underlying biological mechanisms by which inflammation and stress may influence neurogenesis are changes in the kynurenine (KYN) pathway, which is activated under stress. Supplementation with *n*-3 polyunsaturated fatty acids (*n*-3 PUFAs) has anti-inflammatory properties and can increase stress resilience. Whether *n*-3 PUFAs alter KYN stress response is unknown. **Objectives:** This placebo-controlled study investigated the effect of *n*-3 PUFAs on KYN metabolism, inflammation, depressive symptoms, and mood. Moreover, stress-induced changes following a laboratory stressor have been assessed. **Methods:** In this placebo-controlled study, 47 healthy male adults received either 4 g *n*-3 PUFAs per day (Omega-3 group) or a placebo (Placebo group) for 12 weeks. **Results:** A significant group-by-time interaction was found for the inflammatory markers gp130 (F = 7.07, *p* = 0.011), IL-6R alpha (F = 10.33, *p* = 0.003), and TNF_RI (F= 10.92, *p* = 0.002). No significant group-by-time interactions were found for KYN metabolites, depressive symptoms, and mood (except for Hedonic tone (F = 6.50, *p* = 0.014)), nor for stress-induced changes in KYN metabolites and mood following a laboratory stressor. **Conclusions:** Overall, increased *n*-3 PUFA levels in healthy men ameliorate inflammatory markers but do not ameliorate KYN metabolism, depressive symptoms, mood, or KYN metabolism and mood following a stress induction. This study was registered at ClinicalTrials.gov with the identifier NCT05520437 (30/08/2022 first trial registration).

## 1. Introduction

Increased inflammation and increased physiologic stress reactivity are associated with the pathophysiology of mood disorders. Elevations in peripheral blood proinflammatory cytokines, including the interleukin-6 receptor (IL-6R), glycoprotein 130 (gp130), and interleukin-10 (IL-10), are the most reliable biomarkers of increased inflammation in patients with depressive symptoms [1]. Cytokines can cross the blood–brain barrier and impair central neuronal function, which can promote depressive symptoms and low mood [2,3]. The role of proinflammatory cytokines in depressive symptoms is supported by prospective studies that have shown that acute administration of proinflammatory cytokines can trigger depressive symptoms [4,5]. Proinflammatory cytokines have been shown to mediate serotonin deficiency via tryptophan (Trp) degradation by indoleamine 2,3-dioxygenase, resulting in reduced availability of Trp for cerebral serotonin synthesis [5]. Meanwhile, animal models suggest that exposure to stressors facilitates the expression of proinflammatory cytokines and promotes depressive-like behaviour [6]. Physiological responses to acute stressors may vary widely among individuals experiencing the same stressor [7], and reactivity to stressors can be a predictor of depressive symptoms [8]. A meta-analysis has shown that patients with major depressive disorder (MDD) have similar baseline stress cortisol levels to healthy controls, but patients with MDD have significantly higher cortisol levels during the recovery period [9]. While an adaptive stress response is flexible and short-lived, patients with MDD appear to have blunted stress reactivity and impaired recovery from acute stressors [9,10]. An exaggerated, prolonged response to acute stressors may favour a higher increase of proinflammatory cytokines. 

The underlying biological mechanisms by which proinflammatory cytokines and stress can affect neurogenesis are alterations in the kynurenine (KYN) pathway. KYN formation from Trp appears to be activated by psychological or physiological stress as well as directly by inflammatory factors. Proinflammatory cytokines activate the KYN pathway to influence Trp metabolism and secrete neurotoxins that can either decrease serotonin production or promote serotonin reuptake [11,12]. Peripheral Trp conversion to KYN under proinflammatory conditions and under stress has been associated with neuroinflammation and may contribute to depressed mood [13]. In humans, interferon α (IFN-α) therapy showed that an increase in the serum KYN to kynurenic acid (KYNA) ratio is significantly associated with depression severity [14]. 

*N*-3 polyunsaturated fatty acids (*n*-3 PUFAs) may reduce morbidity by regulating inflammation and stress response systems. Higher serum levels of *n*-3 PUFAs appear to reduce the release of proinflammatory cytokines during stress exposure. A randomised controlled trial showed that daily supplementation of 2.5 g of *n*-3 PUFAs (eicosapentaenoic acid (EPA) and docosahexaenoic acid (DHA)) reduced total cortisol and IL-6 during stressors [15]. It has been suggested that supplementation with *n*-3 PUFAs may alter the harmful effects of stress and may thus reduce the risk of depression. *N*-3 PUFAs have anti-inflammatory properties and have a positive effect on brain function [16]. Numerous studies have been conducted on the anti-inflammatory properties of *n*-3 PUFAs on mood. While there is evidence that higher plasma *n*-3 PUFAs levels are associated with improved mood [17], several randomised controlled trials have shown that *n*-3 PUFAs supplementation in healthy individuals has limited effects on mood, either as a main effect or in interaction with other variables, such as stress [18,19]. The conflicting results on the effect of *n*-3 PUFAs supplementation on mood may be due to the poor quality of the methodology. Studies in this area have often implemented supplementation protocols with a duration of less than 3 months [18]—a time that is critical to achieve a significant increase in one of the *n*-3 PUFAs, DHA, in human blood cells. A meta-analysis of twenty-five studies indicated that supplementation with *n*-3 PUFAs reduced depressive symptomology compared to the placebo [20]. However, the quality of evidence from the meta-analysis is insufficient: 25% of the studies included in the meta-analysis were at high risk of bias, and the funnel plot review suggested that the results may be biased [20]. A randomised clinical trial examining the effects of *n*-3 PUFAs supplementation on the risk of later-life depression and mood scores indicated that supplementation with *n*-3 PUFAs did not prevent depression [19]. There was no difference in mood scores between the experimental and control groups and there was a small but statistically significant increase in the risk of depression in those supplementing *n*-3 PUFAs [19].

When investigating biological mechanisms in depressive symptoms, it is important to consider sex-specific differences in inflammatory responses [21,22] and not to generalise conclusions to both sexes. Overall, there are few controlled intervention studies addressing the causal nature of the effect of *n*-3 PUFAs on mood in healthy men, and the results of the few available studies are inconsistent.

To date, no human study has investigated whether *n*-3 PUFAs alter KYN metabolism, and KYN metabolism following a stress induction. The aim of the current study was to investigate the effect of *n*-3 PUFAs supplementation on the regulation of inflammation, KYN metabolism, depressive symptoms, mood, as well as stress-induced changes to the KYN metabolism and mood in healthy men. Although previous studies suggest that the peripheral Trp conversion to KYN activated by stress and inflammatory factors can lead to depressive symptoms, these findings are only based on animal models. Therefore, this is the first study to investigate this phenomenon in humans. In a placebo-controlled design, participants were administered supplementation for 12 weeks. Serum concentrations of metabolites of KYN, including KYN, KYNA, quinolinic acid, xanthurenic acid, picolinic acid, 3-hydroxykynurenine, and 3-hydroxyanthranilic acid, were examined. The inflammatory markers examined include interleukin (IL)-6R alpha, IL-10, gp130, and TNF-RI. The *n*-3 PUFAs supplementation was controlled by investigating the serum concentration levels and percentages of *n*-3 PUFAs. 

## 2. Materials and Methods

### 2.1. Ethics Statement

The study was conducted according to the guidelines of the Declaration of Helsinki and approved by the Bioethics Committee for Research Projects at the University of Gdansk (protocol number 44/2020; date of approval: 20 August 2020. Informed consent was obtained from all participants involved in the study.

### 2.2. Participants

Fifty-one male volunteers aged 23–52 were recruited for this study (Table 1—Characteristics of participants). The age of the participants was chosen based on biological development, which is associated with relatively stable behavioural patterns [23,24]. The majority of participants had a sedentary office job. The maximum amount of moderate physical activity per week was 120 min. Exclusion criteria included DSM-5 psychiatric disorders other than depression and anxiety [25], neurological disorders, chronic illnesses, and any other illnesses that could interfere with the study, vigorous physical activity or moderate physical activity over 120 min per week, and use of dietary supplements containing *n*-3 PUFAs or anti-inflammatory drugs. Participants were instructed not to take any dietary supplements, including *n*-3 PUFAs, or anti-inflammatory drugs during the study period. After the supplementation period, participants were asked to indicate whether they have taken any supplements or medications during the period of the study. Of the fifty-one volunteers who took part in the study, four dropped out after the first intervention period (two from the experimental group and two from the control group). 

### 2.3. Procedure

In a placebo-controlled design, participants received 4000 mg/day of fish oil (EPA 2234 mg; DHA 916 mg) (n = 27) or placebo (MCT) (n = 24) for 12 weeks. Before supplementation, blood samples were taken and mood was assessed using validated psychological measures: Mood Adjective Check List (UMACL) and DASS-21 (t0). At the end of the 12-week supplementation period, mood was assessed and blood samples were taken (t1). A stress response was then induced using a validated stress manipulation test, the Trier Social Stress Test (TSST). After the stress manipulation test, mood was reassessed, and blood samples were taken at two time points: after an acute stressor (t2) and 1 h after an acute stressor (t3). 

### 2.4. Supplements

The dietary supplement Namedsport Omega 3 Double Plus (Namedsport, Lombardy, Italy) was used in this study. The daily dose comprised 3276 mg of n-3 PUFAs, of which there was 2234 mg EPA and 916 mg DHA. The selected DHA and EPA concentration is sufficient to achieve maximum incorporation into erythrocytes within 12 weeks. To ensure construct validity, the supplement chosen in this study is certified by the International Fish Oil Standards™ (IFOS™) program.

Now Foods MCT (medium chain triglyceride) oil was used as a placebo. The daily dose was 4000 mg of MCT. MCT oil was used as a placebo because it does not contain any unsaturated fatty acids. 

### 2.5. Mood Assessment

The UWIST Mood Adjective Check List (UMACL): the Polish adaptation of the UMACL was used to assess mood [26] (Polish adaptation [27]). The questionnaire comprises a list of 29 adjectives. Participants rate on a scale of 1 to 4 the extent to which their present mood corresponds to each of the adjectives. The final score is represented by the three dimensions: energetic arousal (EA), tense arousal (TA), and hedonic tone (TH). A high score for EA corresponds to being restful, energetic, and vigorous; a high score for TA corresponds to being stressed, anxious, or tense; and a high score for TH is associated with being cheerful, satisfied, and happy.

The Depression, Anxiety, and Stress Scale—21 items (DASS-21): the Polish adaptation of DASS-21 was used to assess mood [28]. DASS-21 measures the emotional states of depression, anxiety, and stress (for each of the subscales, the minimum score is 10 and the maximum score is 42, with higher scores representing higher levels of the emotional states).

### 2.6. Stress Manipulation Test

The Trier Social Stress Test (TSST) was used to induce a stress response in participants [29]. The TSST is a three-stage psychosocial stress task conducted in front of a panel of experimenters. It includes (i) a 3 min preparation period, (ii) a 5 min public speaking task, and (iii) a 5 min metal arithmetic task. During the preparation period participants were asked to make an interview-style presentation, which they then presented. In the mental arithmetic task, participants were asked to sequentially subtract the number 7 from a 4-digit number. If the participants made a mistake, the interviewer asked them to start over. The TSST is a reliable method for inducing psychosocial stress [30].

### 2.7. Sample Collection 

The blood samples were filled into 4 mL sodium citrate vacutainer tubes and then centrifuged (4 °C, 4000× *g* for 10 min). Plasma and erythrocytes (RBCs) were collected using a disposable Pasteur pipette and transferred to separate Eppendorf probes and stored in a freezer at −80 °C until further analysis. 

### 2.8. Assessment of Fatty Acids (EPA and DHA)

The serum percentage share and concentration of EPA and DHA in serum were determined by gas chromatography coupled with mass spectrometry. Briefly, total lipids were extracted from the serum samples with a chloroform–methanol mixture (2:1, *v*/*v*) according to Folch et al. [31], and after drying under a nitrogen stream were subjected to hydrolysis with 0.5 M KOH at 90 °C for 3 h. After incubation, the mixtures were acidified with 6 M HCl. A total of 1 mL of water was added, the unesterified fatty acids were extracted three times with 1 mL of n-hexane, and the organic phase was evaporated under a nitrogen stream. The extracts were then derivatised with 10% boron trifluoride in methanol solution at 55 °C for 1.5 h to give fatty acid methyl esters (FAME). Then, 1 mL of water was added and the FAMEs were extracted with 3 × 1 mL of n-hexane, dried under a nitrogen stream, and stored at –20 °C until analysis. 

FAMEs were analysed using a GC-EI-MS QP-2020 NX (Shimadzu, Kyoto, Japan) with chromatographic separation on a Zebron ZB-5MSi capillary column, 30 m × 0.25 mm i.d. × 0.25 μm film thickness (Phenomenex, Torrance, CA, USA). Samples were injected into dichloromethane. A total of 1 μL of the sample was injected in a split mode. The column temperature was set in a range from 60 °C to 300 °C (4 °C/min), with helium as a carrier gas and column head pressure of 60 kPa. The temperatures of the injection, ion source, and transfer line were 300 °C, 200 °C, and 300 °C, respectively. The electron energy used for FAME ionisation was 70 eV; 19-methylarachidic acid was used as an internal standard. Full scan mode was used with mass scan range m/z 45 to 700. Accurate identification of the FA profile, including EPA and DHA, was possible based on FAME mixture standards (Larodan, MI, USA and Merck, Darmstadt, Germany). 

### 2.9. Assessment of Inflammation Markers

Biochemical analysis of IL-6R alpha, IL-10, gp130, and TNF RI levels was performed using high-sensitivity commercially available enzyme-linked immunosorbent assay kits (DRG International, Inc., Springfield, NJ, USA) and Thermo Fisher Scientific Elisa Analyzer (Thermo Fisher Scientific Waltham, MA, USA). 

### 2.10. Assessment of KYN and Its Metabolites

Plasma concentrations of metabolites of KYN metabolism (KYN, KYNA, quinolinic acid, xanthurenic acid, picolinic acid, 3-hydroxykynurenine, and 3-hydroxyanthranilic acid) were determined by high-performance liquid chromatography with tandem mass spectrometry (LC–MS/MS) (Sciex, Framingham, MA, USA), with prior protein precipitation and derivatisation as previously described [32]. An acetonitrile solution of the internal standards was added to the plasma and then vortexed and centrifuged. After drying the supernatant in an airstream, a methanolic solution of hydrochloric acid was added, incubated, and then dried in an airstream. Aqueous formic acid solution was added systematically to the dry residue followed by vortexing and injected into an ExionLC™ (Sciex, Framingham, MA, USA) chromatography equipped with two binary pumps, a degasser, a column oven, and PAL HTC autosampler (CTC Analytics AG, Zwinger, Switzerland) coupled to a 4500 QTrap (Sciex, Framingham, MA, USA) triple quadrupole mass spectrometer.

### 2.11. Statistical Analysis

For every studied outcome variable, we performed a two-way mixed models ANOVA with group (Omega-3/Placebo), time (before/after supplementation), and the groupxtime interaction as fixed effects and participant as random effect. Additionally, for variables measured before and after the stress task, we performed a similar two-way mixed models ANOVA (with time effects before, after, and 1 h after stress task). If one or more effects were statistically significant, we conducted a post hoc analysis with pairwise comparisons of estimated marginal means. We corrected for multiple comparisons with Tukey HSD correction. For all analyses, results were deemed statistically significant if *p* < 0.05. Data analysis was carried out in Python (v.3.11) using packages Numpy (v.1.26) [33], Pandas (v.2.2) [34], and Pymer4 (v.0.8) [35]. Plots were created with Matplotlib (v.3.8) [36] and Seaborn (v.0.13) [37].

## 3. Results

### 3.1. Fatty Acids Profile

**Figure 1:**
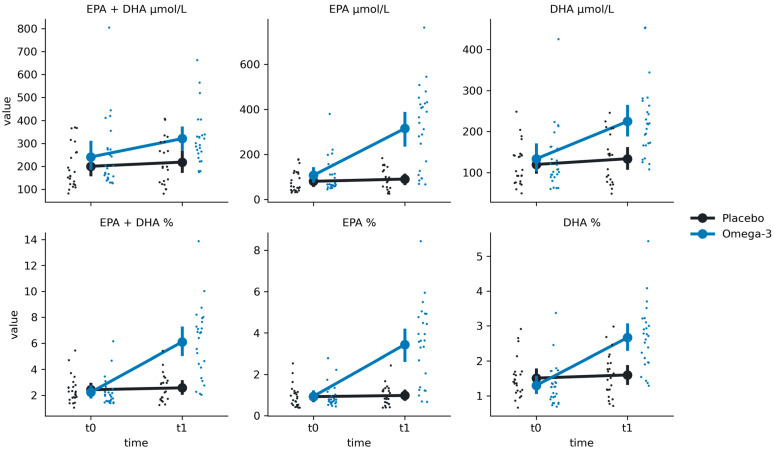
Distributions of the group x time interaction effect on the fatty acids profiles in the Placebo (black) and Omega-3 (blue) groups at times t0 (before supplementation) and t1 (after supplementation). Large points represent mean values, with error bars representing a 95% confidence interval around the mean value. Small points represent single observations.

For EPA %, we have found a significant group-by-time interaction (F(1, 49.29) = 34.94, *p* < 0.001) (Figure 1; Table 2). Post hoc comparisons revealed significantly higher EPA % values at t1 in comparison to t0 for the Omega-3 group (Mt0 = 0.94 ± 0.55, Mt1 = 3.44 ± 1.93, t = −8.76, *p* < 0.001) but not for the Placebo group (Mt0 = 0.92 ± 0.54, Mt1 = 0.98 ± 0.5, t = −0.17, *p* = 0.86). Similarly, we found a group-by-time interaction for EPA μmol/L (F(1, 47.12) = 36.84, *p* < 0.001). Post hoc comparisons revealed significantly higher EPA μmol/L values at t1 in comparison to t0 for the Omega-3 group (Mt0 = 107.37 ± 76.18, Mt1 = 314.83 ± 180.14, t = −7.23, *p* < 0.001) but not for the Placebo group (Mt0 = 80.49 ± 47.72, Mt1 = 90.4 ± 47.43, t = −0.17, *p* = 0.86).

Similarly, a group-by-time interaction was found for DHA % (F(1, 43.46) = 10.62, *p* = 0.002). Post hoc comparisons revealed significantly higher DHA μmol/L values at t1 in comparison to t0 for the Omega-3 group (Mt0 = 133.74 ± 76.82, Mt1 = 224.74 ± 90.09, t = −5.50, *p* < 0.001) but not for the Placebo group (Mt0 = 120.1 ± 50.09, Mt1 = 134.04 ± 60.03, t = −0.74, *p* = 0.46). The same interaction was also found for DHA μmol/L (F(1, 43.46) = 10.62, *p* = 0.002). Post hoc comparisons revealed significantly higher DHA μmol/L values at t1 in comparison to t0 for the Omega-3 group (Mt0 = 133.74 ± 76.82, Mt1 = 224.74 ± 90.09, t = −5.50, *p* < 0.001) but not for the Placebo group (Mt0 = 120.1 ± 50.09, Mt1 = 134.04 ± 60.03, t = −0.74, *p* = 0.46).

As expected, the results for EPA + DHA % also revealed a significant group-by-time interaction (F(1, 49.89) = 19.63, *p* < 0.001), with the Omega-3 group having higher values at t1 than at t0 (Mt0 = 2.24 ± 1.09, Mt1 = 6.1 ± 2.79, t = −9.30, *p* < 0.001) and no differences in the Placebo group (Mt0 = 2.43 ± 1.04, Mt1 = 2.58 ± 1.07 ± 50.09, t = −0.29, *p* = 0.77). Finally, the same pattern was found for EPA + DHA μmol/L, with a significant interaction (F(1, 48.49) = 5.77, *p* = 0.02) and time differences in the Omega-3 group (Mt0 = 241.11 ± 149.37, Mt1 = 320.85 ± 120.01, t = −5.19, *p* < 0.001) but not in the Placebo group (Mt0 = 200.58 ± 91.96, Mt1 = 218.58 ± 98.76, t = −0.72, *p* = 0.48).

### 3.2. KYN Pathway

**Figure 2:**
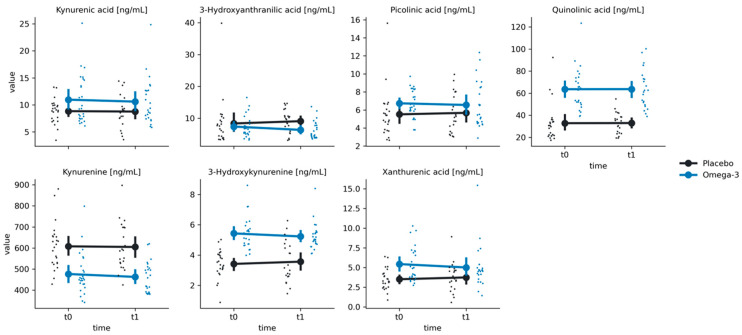
Distributions of the group x time interaction effect on the kynurenine pathway in the Placebo (black) and Omega-3 (blue) groups at times t0 (before supplementation) and t1 (after supplementation). Large points represent mean values, with error bars representing a 95% confidence interval around the mean value. Small points represent single observations.

**Figure 3:**
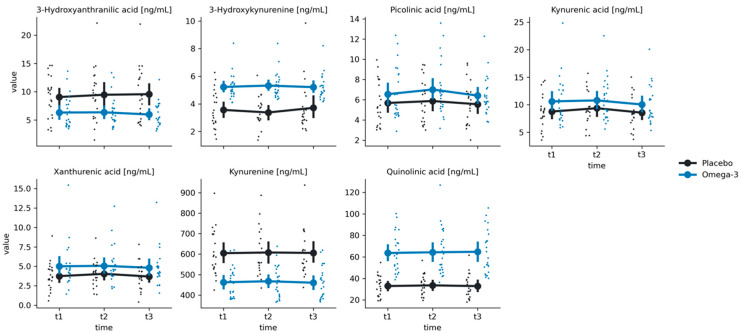
Distributions of the group x time interaction effect on the KYN pathway in the Placebo (black) and Omega-3 (blue) groups at times t1 (after supplementation), t2 (straight after stress induction), and t3 (one hour after stress induction). Large points represent mean values, with error bars representing a 95% confidence interval around the mean value. Small points represent single observations.

There were significant group effects for all KYN metabolites (all *p*-values < 0.01); however, we have not found any significant time effects nor group-by-time interactions (all *p*-values > 0.05) (Figure 2 and Figure 3; Table 2).

### 3.3. Inflammation Markers

**Figure 4:**
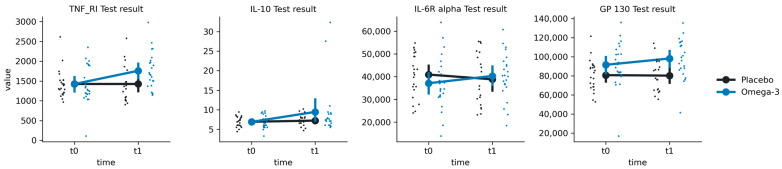
Distributions of the group x time interaction effect on inflammation markers in the Placebo (black) and Omega-3 (blue) groups at times t0 (before supplementation) and t1 (after supplementation). Large points represent mean values, with error bars representing a 95% confidence interval around the mean value. Small points represent single observations.

For IL-10, we have found a significant main effect of time (F(1, 28.3) = 4.30, *p* = 0.047) but not group or group-by-time interaction (*p*-values > 0.05) (Figure 4; Table 2). Post hoc comparisons revealed significantly higher IL-10 at t1 compared to t0 (Mt0 = 6.89 ± 1.61, Mt1 = 9.44 ± 6.66, t = −2.72, *p* = 0.011) in the Omega-3 group but not in the Placebo group (Mt0 = 6.92 ± 1.3, Mt1 = 7.22 ± 1.53, t = −0.302, *p* > 0.05). For GP 130, we have found both a main effect of group (F(1, 46.2) = 6.57, *p* = 0.014) and a group-by-time interaction (F(1, 39.4) = 7.07, *p* = 0.011). Post hoc comparisons revealed significantly higher GP 130 results at t1 compared to t0 in the Omega-3 group (Mt0 = 91,537 ± 22,774, Mt1 = 98,177 ± 20,706, t = −3.28, *p* = 0.002) but not in the Placebo group (Mt0 = 80,718 ± 16,218, Mt1 = 80,225 ± 17,079, t = 0.58, *p* = 0.56). For IL-6R alpha, we have found a significant group-by-time interaction (F(1, 39.5) = 10.33, *p* = 0.003), with post hoc tests showing an increase in the Omega-3 group (Mt0 = 37,097 ± 11,295, Mt1 = 40,293 ± 10,220, t = 3.54, *p* = 0.001) but not in the Placebo group (Mt0 = 40,901 ± 9695, Mt1 = 38,815 ± 11,586, t = 1.09, *p* = 0.28). For TNF-RI, we found both a main effect of time (F(1, 42.4) = 7.88, *p* = 0.007) and a group-by-time interaction (F(1, 42.4) = 10.92, *p* = 0.002). Post hoc tests revealed an increase in TNF-RI in the Omega-3 group (Mt0 = 1424 ± 464, Mt1 = 1759 ± 465, t = −4.49, *p* < 0.001) but not in the Placebo group (Mt0 = 1426 ± 351, Mt1 = 1426 ± 458, t = 0.34, *p* = 0.74).

### 3.4. Depressive Symptoms and Mood Measures 

**Figure 5:**
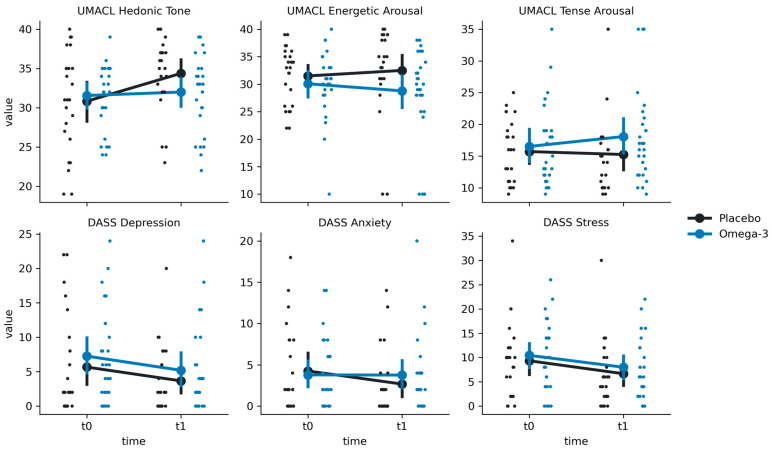
Distributions of the group x time interaction effect on mood and depressive symptoms in the Placebo (black) and Omega-3 (blue) groups at times t0 (before supplementation) and t1 (after supplementation). Large points represent means, with error bars representing a 95% confidence interval around the mean. Small points represent single observations.

For DASS, we have found significant effects of time in the STRESS scale (F(1, 47.9) = 6.93, *p* = 0.011) and in the DEPRESSION scale (F(1, 47.7) = 5.20, *p* = 0.027). However, there were no significant group-by-time interactions (all *p*-values > 0.05), suggesting that *n*-3 PUFAs supplementation had no effect on DASS outcomes (Figure 5; Table 2). For UMACL TH, we found a significant time effect (F(1, 49) = 10.77, *p* = 0.002) and a significant group-by-time interaction (F(1, 49) = 6.50, *p* = 0.014). Post hoc tests indicated that there was an increase in scores from t0 to t1 in the Placebo group (Mt0 = 30.83 ± 6.38, Mt1 = 34.38 ± 4.69; t = −4.00; *p* < 0.001) but not in the Omega-3 group (Mt0 = 31.56 ± 4.25, Mt1 = 32.0 ± 4.93; t = −0.53, *p* = 0.60). No significant differences were found for other UMACL subscales (*p* > 0.05).

### 3.5. Stress Induction 

Among the KYN metabolites, we have found significant group effects for 3-Hydroxykynurenine (F(1, 45.7) = 32.83, *p* < 0.001), KYN (F(1, 45.04) = 26.94, *p* < 0.001), quinolinic acid (F(1, 45.12) = 46.82, *p* < 0.001), xanthurenic acid (F(1, 45.01), *p* = 0.046), and 3-Hyrdroxyanthranilic acid (F(1, 44.96) = 10.32, *p* = 0.002) (Table 2). Additionally, we found a significant time effect for KYNA (F(2, 84.1) = 10.30, *p* < 0.001). Post hoc comparisons revealed significant lowering of KYNA levels between t2 and t3 for both the Omega-3 group (Mt2 = 10.78 ± 3.78, Mt3 = 10.03 ± 3.43, t = 3.38, *p* = 0.003) and the Placebo group (Mt2 = 9.35 ± 3.05, Mt3 = 8.54 ± 2.91, t = 2.96, *p* = 0.011). Additionally, for the Omega-3 group, KYNA levels were significantly lower in t3 in comparison to t1 (Mt1 = 10.6 ± 4.29, Mt2 = 10.03 ± 3.43, t = 3.08, *p* = 0.008). However, we did not find any significant group-by-time interactions for any of the KYN metabolites (all *p* values > 0.05).

We did not find effects of stress induction on UMACL outcomes (all *p*-values > 0.05).

## 4. Discussion

The changes in the fatty acid profile after 3 months of supplementation showed that the supplementation significantly increased plasma EPA and DHA concentrations in the Omega-3 group but not in the Placebo group. However, no significant treatment effects were found for any of the measures of the KYN pathway and depressive symptoms, and majority of the measures of mood. Thus, it can be concluded that the study provides no evidence that increasing *n*-3 PUFAs status in healthy men by taking *n*-3 PUFAs supplements has any benefit on the KYN pathway, mood, depressive symptoms, or KYN pathway and mood following a stress induction. 

### 4.1. n-3 PUFAs and KYN Pathway

The main observation of this placebo-controlled experiment is that increasing *n*-3 PUFA levels in healthy men had no effect on any of the KYN metabolites. One possible explanation for the null effect of *n*-3 PUFAs supplementation on KYN metabolites is a potential role of a moderating effect of physical activity on the relationship between the KYN metabolic pathway and the fatty acids profile. Results from a randomised controlled trial suggest that *n*-3 PUFAs supplementation accompanied by endurance training leads to increased plasma concentrations of neurotoxic 3-hydroxykynurenine and its formation of the neuroprotective metabolite, picolinic acid. *N*-3 PUFAs had no effect on the KYN pathway in a group of inactive participants [32].

When drawing conclusions on *n*-3 PUFAs and the KYN pathway, it is important to consider group differences—there were significant group effects for all KYN metabolites. Despite the group differences, the distributions showed similar variance, so it seemed appropriate to compare the two groups. The study also tested for an interaction effect, which can be detected independently of group effects. This allows comparison of the two groups independent of the differences between the groups. 

### 4.2. n-3 PUFAs, Depressive Symptoms, and Mood 

In the current study, no significant differences were observed in longitudinal mood scores when comparing the Omega-3 group with the Placebo group, with the exception of the ‘hedonic tone’ subscale. Increased ‘hedonic tone’ scores after 12 weeks of *n*-3 PUFA supplementation in the Placebo group but not in the *n*-3 PUFA group is an unexpected finding. Hedonic tone is the trait underlying one’s characteristic ability to feel pleasure, and lower scores represent a reduced capacity to experience pleasure [27]. It can therefore be concluded that the Placebo group improved their mood on this one dimension. However, given the relatively large number of variables in the study, it cannot be ruled out that the result is due to chance.

While the results of the current study are contrary to some evidence for a mood-enhancing effect, they are consistent with the results of a meta-analysis that showed no benefit of *n*-3 PUFAs supplementation on depressive symptoms in community-based samples of adults without clinical depression at baseline [38]. A possible explanation for the null effects of *n*-3 PUFAs supplementation on mood may be the sample of participants recruited in the current study. None of the participants suffered from MDD, and a majority of the participants scored ‘normal’ on the DASS-21. Considering that participants scored low on all subscales of DASS-21 and scored low on Tense Arousal and high on Energetic Arousal and Hedonic Tone UMACL subscales at baseline, the effects of *n*-3 PUFAs supplementation may have been very subtle and not detected. Our findings are consistent with the results of the largest randomised clinical trial (N = 18,353) showing that *n*-3 PUFAs do not prevent depressive symptoms or improve mood [19]. In this randomised trial, participants without clinically relevant depressive symptoms at baseline were randomly assigned to supplement with *n*-3 PUFAs /placebo over a 5-year treatment period. 

When drawing conclusions from the study, it should be noted that the study sample consisted of a general adult population without clinically relevant depressive symptoms at baseline. The aim of this study was to investigate the effect of *n*-3 PUFAs supplementation on mood, including depressive symptoms, in healthy male adults. Hence, the conclusions from this study can only be applied to individuals without mental health problems. It cannot be extrapolated to the clinical population of patients with clinically diagnosed MDD. It is possible that a sample of patients with MDD would have yielded different results. A meta-analysis of the effects of *n*-3 PUFAs on depressed mood indicated that individuals with a diagnosed depressive disorder may benefit to some extent from *n*-3 PUFAs supplementation, whereas no evidence of benefit was found in individuals without a diagnosis of depressive disorder. In contrast, there is some evidence for the efficacy of *n*-3 PUFAs on mood in the general population. For example, the study which was conducted using a subset of data from ‘The Hordaland Health Study ‘97–‘99’ and was a cross-sectional population-based health survey in Norway showed that regular intake of cod liver oil was negatively associated with high levels of depressive symptoms in the general population [39]. This study further indicated that the risk of depressive symptoms decreased over time depending on the duration of cod liver oil supplementation, i.e., participants who had been supplementing cod liver oil for 9–12 months had fewer depressive symptoms than participants who had been supplementing cod liver oil for 1–4 months. Thus, it appears that the duration of *n*-3 PUFAs supplementation may be crucial when investigating mood. While a supplementation of 12 weeks is justified as sufficient to achieve a significant increase in one of the *n*-3 PUFAs, DHA, in human blood cells, more time appears to be required to detect changes in mood. 

### 4.3. n-3 PUFAs and Inflammation 

Supplementation with *n*-3 PUFAs was successful in increasing neuroprotective factors, including TNF-RI, IL-6R alpha, and gp130. After the supplementation period, significantly higher levels of IL-10 were found in the Omega-3 group. When drawing conclusions about this effect, it should be noted that this result should be taken with caution. Visual inspection of individual cases showed that the Omega-3 group contained two outliers, which could have caused the observed interaction effect.

The findings on increased levels of TNF-RI, IL-6R alpha, and gp130 are consistent with previous studies. IL-6R alpha has a direct binding with the membrane-bound gp130, which is a protein receptor with neuroprotective abilities. IL-6R alpha plays a critical role in mediating the anti-inflammatory response by interacting with the gp130 protein [40,41]. While IL-6 can have a negative effect on mood, gp130 can counteract depressive symptoms. There is in vitro and clinical evidence that *n*-3 PUFAs protect against inflammation through the production of LOX and CYP450 lipid mediators [42]. These lipid mediators seem to support the antidepressant, anti-inflammatory, and neuroprotective effects of *n*-3 PUFAs.

### 4.4. n-3 PUFAs and KYN Metabolism and Mood Following a Stress Induction

The experiment found no differences in KYN metabolism between the experimental and control groups following a stress induction. Although there are few studies on the effects of acute stress on KYN metabolism in humans, an animal model suggests that physical activity may play a moderating role in this context [43]. Based on the animal model, it seems that PGC-1α1 in skeletal muscle modulates the KYN metabolic pathway and mediates resilience to stress-induced depression. There is evidence of a differential effect of *n*-3 PUFAs on the KYN pathway in physically active and physically inactive men. In a randomised clinical trial, *n*-3 PUFAs had no effect on the KYN pathway in inactive participants but had a positive effect on the KYN pathway in amateur endurance runners [32]. Considering a possible moderating effect of physical activity on the KYN pathway, future research should investigate the relationship between acute stress, KYN pathway, and physical activity. This could potentially extend the animal models.

### 4.5. Strengths of the Study

Our study is the first placebo-controlled trial to investigate the effect of *n*-3 PUFAs supplementation on the KYN pathway following a stress induction. Among the many strengths of this placebo-controlled study is the 12-week supplementation period—a period that is crucial to achieve a significant increase in one of the *n*-3 PUFAs, DHA, in human blood cells. To ensure construct validity, participants received *n*-3 PUFAs supplements certified by the International Fish Oil Standards™ (IFOS™) programme. This is particularly important because IFOS verifies the contents of each *n*-3 PUFAs against the manufacturer’s declarations, which ensures construct validity. In the current study, MCT was used as a placebo—both MCT and *n*-3 PUFAs were administered in the form of similar looking capsules. Plasma EPA and DHA concentrations were also measured to investigate whether *n*-3 PUFAs supplementation was successful. In addition, all psychological tools applied in the following study are valid and reliable. 

### 4.6. Limitations of the Study

Some limitations of the study included methodological limitations of the materials used. Although the TSST is a validated tool to induce stress in participants, the standardised procedure of stress induction does not allow for changing the tasks when stress is induced at different time points. Stress induction with the TSST at two different time points (i.e., before and after the intervention) would require the use of the same procedures, so the stressor may have been perceived as less stressful at the second time point [44]. For this reason, each participant completed the acute laboratory stressor only after the intervention. We were therefore unable to adjust for baseline reactivity to account for possible interindividual variability. The decision to induce stress at only one time point was based on the familiarity with the laboratory stressor. Nevertheless, there may have been individual differences in the participants’ experience of stress. To control for individual differences in stress experience, it would have been useful to investigate subjective feelings towards the stress induction test. The UMACL was applied to measure mood. This tool measures only some dimensions of mood—i.e., EA, TA, and TH. 

Although the UMACL can be used as a repeated measure, it may not be sensitive to minor changes in mood [27]. Furthermore, the study followed a 12-week protocol, which was enough time to achieve a significant increase in one of the *n*-3 PUFAs, DHA, in human blood cells. However, it is likely that a longer protocol should have been conducted to achieve a change in depressive symptoms [39]. 

Finally, the study only examined men, and hence the results cannot be generalised to both male and female adults. Inflammatory responses differ between men and women [21,22]. It is therefore important not to generalise the conclusions to both sexes. Based on previous findings that inflammatory responses are more pronounced in women than in men [21,22], it is possible that *n*-3 PUFAs may have a stronger effect on the KYN pathway in women. 

### 4.7. Implications

The Academy of Nutrition and Dietetics recommends at least 500 mg/day of EPA and DHA for adults [45]. The health benefits promoted include mental health. Our results suggest that daily *n*-3 PUFAs supplementation alone may not have clinical benefit in improving the KYN pathway and mood in a non-clinical group of men without a diagnosis of MDD.

## 5. Conclusions

Combining experimental and longitudinal methodologies, this study found no evidence that increasing *n*-3 PUFA status in healthy men by taking *n*-3 PUFAs supplements has any benefit on the KYN pathway, mood, depressive symptoms, or KYN metabolism following a stress induction. The clinical benefit of supplementation of *n*-3 PUFAs in healthy men to improve the KYN pathway and mood is therefore questionable. When drawing conclusions from the study, it is important to consider that the study group consisted of healthy men rather than men with MDD. Future research on the effect of *n*-3 PUFAs on KYN metabolism and mood should extend the animal models by further investigating clinical samples of patients with MDD in this context. 

## Figures and Tables

**Table 1:** Characteristics of participants (Omega-3 and Placebo groups).

Sample Characteristics	Omega-3 (*n* = 25)	Placebo (*n* = 22)
	M ± SD	M ± SD
Age	36.04 years ± 5.43	36.79 ± 7.17
Weight [kg]	92.38 ± 13.79	86.82 ± 16.06
BMI	28 ± 3.89	26.78 ± 4.41

**Table 2:** Means, standard averages, and analysis of variance results for fatty acids profile, kynurenine pathway, inflammation markers, mood, and depressive symptoms.

Group	Omega-3				Placebo				Group Main Effect		Time Main Effect		Group x Time Interaction	
Time	t0	t1	t2	t3	t0	t1	t2	t3	F-Statistic	*p*-Value	F-Statistic	*p*-Value	F-Statistic	*p*-Value
3-Hydroxyanthranilic acid [ng/mL]	7.37 ± 3.37	6.33 ± 2.85	6.37 ± 2.67	5.99 ± 2.15	8.35 ± 7.47	9.07 ± 3.6	9.47 ± 4.63	9.58 ± 4.56	10.33	0.002 *	0.16	0.85	0.41	0.66
3-Hydroxykynurenine [ng/mL]	5.43 ± 1.05	5.23 ± 0.9	5.32 ± 0.91	5.21 ± 0.9	3.41 ± 0.93	3.56 ± 1.34	3.38 ± 1.12	3.71 ± 1.81	32.83	<0.001 *	0.38	0.68	1.38	0.26
Kynurenic acid [ng/mL]	10.96 ± 4.56	10.6 ± 4.29	10.78 ± 3.78	10.03 ± 3.43	8.83 ± 2.23	8.75 ± 3.0	9.35 ± 3.05	8.54 ± 2.91	5.23	0.026 *	0.09	0.76	0.01	0.92
Kynurenine [ng/mL]	476.18 ± 100.04	462.48 ± 73.25	467.45 ± 74.62	460.38 ± 77.04	607.66 ± 109.67	604.7 ± 112.35	608.08 ± 117.59	605.84 ± 111.5	26.95	<0.001 *	1.78	0.18	0.27	0.76
Picolinic acid [ng/mL]	6.74 ± 1.54	6.55 ± 2.55	7.01 ± 2.56	6.41 ± 1.91	5.51 ± 2.67	5.68 ± 2.17	5.87 ± 2.18	5.55 ± 2.16	4.39	0.04 *	0.001	0.98	0.17	0.68
Quinolinic acid [ng/mL]	63.6 ± 18.47	63.68 ± 17.91	64.3 ± 21.37	64.75 ± 20.29	32.82 ± 17.07	32.95 ± 9.49	33.58 ± 9.81	32.89 ± 11.04	58.56	<0.001 *	0.001	0.98	0.018	0.89
Xanthurenic acid [ng/mL]	5.44 ± 2.18	5.01 ± 2.73	5.07 ± 2.35	4.82 ± 2.34	3.51 ± 1.34	3.75 ± 1.79	4.04 ± 1.7	3.7 ± 1.7	10.5	0.002 *	0.03	0.85	0.73	0.40
EPA μmol/L	107.37 ± 76.18	314.83 ± 180.14			80.49 ± 47.72	90.4 ± 47.43	80.49 ± 47.72	90.4 ± 47.43	33.20	<0.001 *	27.17	<0.001 *	22.52	<0.001 *
EPA %	0.94 ± 0.55	3.44 ± 1.93			0.92 ± 0.54	0.98 ± 0.5	0.92 ± 0.54	0.98 ± 0.5	28.38	<0.001 *	37.91	<0.001 *	34.93	<0.001 *
DHA %	1.3 ± 0.59	2.66 ± 0.94			1.51 ± 0.56	1.6 ± 0.63	1.51 ± 0.56	1.6 ± 0.63	6.91	0.011 *	47.21	<0.001 *	37.84	<0.001 *
DHA μmol/L	133.74 ± 76.82	224.74 ± 90.09			120.1 ± 50.09	134.04 ± 60.03	120.1 ± 50.09	134.04 ± 60.03	10.33	0.002 *	18.80	<0.001 *	10.62	0.002 *
EPA + DHA %	2.24 ± 1.09	6.1 ± 2.79			2.43 ± 1.04	2.58 ± 1.07	2.43 ± 1.04	2.58 ± 1.07	19.64	<0.001 *	43.94	<0.001 *	38.45	<0.001 *
EPA + DHA μmol/L	241.11 ± 149.37	320.85 ± 120.01			200.58 ± 91.96	218.58 ± 98.76	200.58 ± 91.96	218.58 ± 98.76	5.77	0.020 *	16.90	<0.001 *	9.45	0.004 *
GP 130	91,537.61 ± 22,774.97	98,177.17 ± 20,706.59			80,718.89 ± 16,218.94	80,225.42 ± 17,079.21	80,718.89 ± 16,218.94	80,225.42 ± 17,079.21	6.57	0.014 *	3.30	0.08	7.07	0.011 *
IL-10	6.89 ± 1.61	9.44 ± 6.66			6.92 ± 1.3	7.22 ± 1.53	6.92 ± 1.3	7.22 ± 1.53	1.90	0.18	4.30	0.047 *	2.66	0.11
IL-6R alpha	37,097.35 ± 11,295.89	40,293.76 ± 10,220.24			40,901.47 ± 9695.93	38,815.35 ± 11,586.19	40,901.47 ± 9695.93	38,815.35 ± 11,586.19	0.21	0.64	2.60	0.12	10.33	0.003 *
TNF-RI	1424.8 ± 464.83	1759.74 ± 465.68			1426.31 ± 351.59	1426.6 ± 458.39	1426.31 ± 351.59	1426.6 ± 458.39	2.26	0.14	7.88	0.007 *	10.92	0.002 *
UMACL Energetic Arousal	30.07 ± 6.06	28.78 ± 7.81	30.3 ± 7.42		31.5 ± 5.27	32.5 ± 8.02	32.33 ± 8.08		2.56	0.12	0.02	0.89	1.15	0.29
UMACL Tense Arousal	16.52 ± 6.59	18.07 ± 7.36	16.81 ± 6.73		15.71 ± 4.92	15.25 ± 7.11	16.04 ± 6.9		1.43	0.23	0.27	0.61	0.91	0.34
UMACL Hedonic Tone	31.56 ± 4.25	32.0 ± 4.93	32.44 ± 4.96		30.83 ± 6.38	34.38 ± 4.69	34.25 ± 4.61		0.41	0.52	10.77	0.002 *	6.50	0.014 *
DASS Anxiety	3.78 ± 4.24	3.76 ± 4.59			4.25 ± 5.12	2.67 ± 3.94			0.07	0.79	2.27	0.14	2.24	0.14
DASS Depression	7.26 ± 6.73	5.2 ± 6.38			5.67 ± 7.29	3.67 ± 4.89			1.07	0.31	5.20	0.027 *	0.004	0.95
DASS Stress	10.44 ± 7.05	8.0 ± 6.45			9.33 ± 7.86	6.67 ± 6.66			0.49	0.49	6.94	0.011 *	0.01	0.92

* means the result is statistically significant.

## Data Availability

Data will be shared upon reasonable request. To request data from the study please email the principal investigator: monika.bidzan@gumed.edu.pl.
